# Functional expression cloning identifies COX-2 as a suppressor of antigen-specific cancer immunity

**DOI:** 10.1038/cddis.2014.531

**Published:** 2014-12-11

**Authors:** C Göbel, F Breitenbuecher, H Kalkavan, P S Hähnel, S Kasper, S Hoffarth, K Merches, H Schild, K S Lang, M Schuler

**Affiliations:** 1 Department of Medical Oncology, West German Cancer Center, University Hospital Essen, University Duisburg-Essen, Essen 45122, Germany; 2 Department of Immunology, University Hospital Essen, University Duisburg-Essen, Essen 45122, Germany; 3 Institute for Immunology, University Medical Center, Mainz 55101, Germany; 4 German Cancer Consortium (DKTK), Heidelberg 69120, Germany

## Abstract

The efficacy of immune surveillance and antigen-specific cancer immunotherapy equally depends on the activation of a sustained immune response targeting cancer antigens and the susceptibility of cancer cells to immune effector mechanisms. Using functional expression cloning and T-cell receptor (TCR) transgenic mice, we have identified cyclooxygenase 2/prostaglandin-endoperoxide synthase 2 (COX-2) as resistance factor against the cytotoxicity induced by activated, antigen-specific T cells. Expressing COX-2, but not a catalytically inactive COX-2 mutant, increased the clonogenic survival of E1A-transformed murine cancer cells when cocultured with lymphocytes from St42Rag2^−/−^ mice harboring a transgenic TCR directed against an E1A epitope. COX-2 expressing tumors established in immune-deficient mice were less susceptible to adoptive immunotherapy with TCR transgenic lymphocytes *in vivo*. Also, immune surveillance of COX-2-positive tumor cells in TCR transgenic mice was less efficient. The growth of murine MC-GP tumors, which show high endogenous COX-2 expression, in immunocompetent mice was effectively suppressed by treatment with a selective COX-2 inhibitor, celecoxib. Mechanistically, COX-2 expression blunted the interferon-gamma release of antigen-specific T cells exposed to their respective cellular targets, and increased the expression of interleukin-4 and indoleamine 2,3-dioxygenase by tumor cells. Addition of interferon-gamma sensitized COX-2 expressing cancer cells to tumor suppression by antigen-specific T cells. In conclusion, COX-2, which is frequently induced in colorectal cancer, contributes to immune evasion and resistance to antigen-specific cancer immunotherapy by local suppression of T-cell effector functions.

Anticancer immunity mediates immune surveillance and may be exploited for cancer immunotherapy. It involves innate immunity and natural killer cells, and antigen-specific immunity directed against cancer-specific antigens and viral antigens. Several escape mechanisms from cancer-specific immune surveillance and immunotherapy have been described. These comprise defective antigen processing and presentation by downmodulation of major histocompatibility complex (MHC) expression as well as immune editing of the antigen repertoire of a given cancer.^1^ Upregulated inhibitory ligands, such as PD-L1, and secreted factors like indoleamine 2,3-dioxygenase (IDO, encoded by *IDO1*) functionally suppress antigen-presenting cells and cytotoxic cellular immune effectors.^2, 3^ In addition, cell-autonomous mechanisms may decrease susceptibility of cancer to immune effector mechanisms. These involve granule-dependent cytotoxicity involving perforin and granzymes, death receptor-induced apoptosis, complement-dependent cytotoxicity and secreted factors such as interferons, all of which trigger specific intracellular death pathways.^4, 5, 6, 7, 8^ Accordingly, the success of immune prevention and immunotherapy relies on both, the activation of a potent immune response against cancer and its susceptibility to immune elimination.

Clinically applied modalities of cancer immunotherapy include the adoptive transfer of cellular immune effectors by means of allogeneic stem cell transplantation and donor lymphocyte therapy, monoclonal antibodies with direct and indirect cytotoxic mechanisms, and active immunotherapy with cellular and acellular vaccines.^9^ Moreover, immune regulatory interventions using cytokines and, more recently, immune regulatory antibodies directed against CTLA-4, PD-1 and PD-L1 have been employed with varying success.^10, 11^ A detailed understanding of the activation and regulation of a cancer-specific immune reaction as well as the determinants of efficacy of the effector phase of immune elimination is crucial for successful implementation and improvement of such immunotherapies. To this end we have developed experimental systems for unbiased identification of cell-autonomous mechanisms that modulate the susceptibility of cancer to the cytotoxic effects of activated, antigen-specific T cells. We identify cyclooxygenase 2/prostaglandin-endoperoxide synthase 2 (COX-2), a pathogen-induced enzyme involved in prostaglandin synthesis, as mediator of resistance to the effector phase of antigen-specific cancer immunity. Deregulation of COX-2 has been implied in the pathogenesis of several cancers, in particular colorectal cancer, where it impacts on oncogenic signaling, invasion and metastasis, survival and angiogenesis.^12, 13, 14, 15^ Moreover, COX-2-dependent prostaglandin release can suppress antigen presentation and immune activation in cancer.^16^ Here we describe COX-2 as a suppressor of antigen-induced interferon-gamma secretion of T cells and inducer of immunosuppressive factors that contributes to escape from immune surveillance and resistance to cellular immunotherapy. COX-2 may serve as predictive biomarker and as therapeutic target for modulation of immune resistance in cancer.

## Results

### Identification and validation of COX-2 as resistance factor to tumor suppression by antigen-specific T cells

Cytotoxic T cells directed against antigens that are endogenously expressed and presented by cancer cells are critically involved in antigen-specific cancer immunotherapy.^17^ In order to study mechanisms modulating the effector phase of antigen-specific cellular immunotherapy we have established experimental systems based on St42 mice, which express a transgenic T-cell receptor (TCR) recognizing the cognate peptide SGPSNTPPEI from the adenovirus E1A protein (amino acids 234–243) when presented by H2-D^b^ (Toes *et al.*^18^). St42 mice were bred on a Rag2^−/−^ background to obtain homozygous St42Rag2^−/−^ mice as source of E1A-specific cytotoxic T cells. Murine embryonic fibroblasts (MEF) from C57BL/6 mice were transformed by expression of oncogenic H-Ras in combination with adenoviral E1A (E1A-MEF) or Myc (Myc-MEF) to serve as H2-D^b^ positive cancer models. Using this system we have conducted unbiased functional screens to identify factors that confer resistance to cancer cell elimination by antigen-specific cytotoxic T cells.

**Figure 1:**
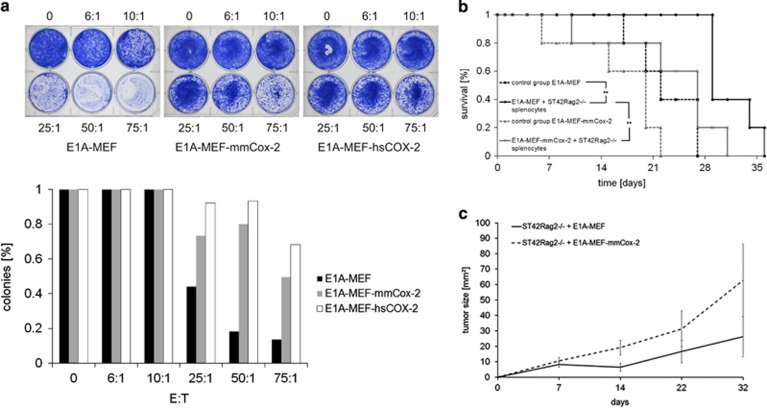
COX-2 confers resistance against tumor suppression by antigen-specific T cells *in vitro* and *in vivo.* (**a**) Clonogenic survival of E1A-MEF, and E1A-MEF expressing the murine (mmCox-2) or human (hsCOX-2) following incubation with St42Rag2^−/−^ splenocytes directed against the E1A (amino acids 234–243) epitope at the indicated effector-to-target ratios (6 : 1 to 75 : 1). Representative photographs (upper panel) and enumeration of clones (mean values) of three independent experiments each are shown (lower panel). (**b**) Survival of untreated NOD/SCID mice bearing E1A-MEF fibrosarcomas (closed boxes/dashed line), or E1A-Cox2-MEF fibrosarcomas (closed triangles/dashed line), and E1A-MEF fibrosarcoma-bearing mice (closed circles/solid line) or E1A-Cox2-MEF fibrosarcoma-bearing mice (crosses/solid lines) both treated with a single adoptive transfer of 3.8 × 10^7^ St42Rag2^−/−^ splenocytes. Kaplan–Meier survival plots (five mice per group; *HR=0.34, *P*=0.0347; **HR=0.23, *P*=0.0027, log-rank test). (**c**) Tumor growth of E1A-MEF (solid line) and E1A-Cox2-MEF (dashed line) injected in St42Rag2^−/−^ mice. Mean values (±S.E.M.) of five mice per group are given

Long-term clonogenic survival of E1A-MEF incubated with splenocytes from St42Rag2^−/−^ mice was significantly reduced when compared with Myc-MEF incubated with St42Rag2^−/−^ splenocytes, or E1A-MEF cultured in the absence of immune effectors (Supplementary Figure 1). This result confirmed that E1A-MEF, but not Myc-MEF, present endogenously processed E1A antigens and are thus susceptible to the H2-D^b^-restricted cytotoxic effects of TCR transgenic St42Rag2^−/−^ splenocytes. Next, we applied a bicistronic retroviral vector system to express a human cancer cDNA library^19^ and the reporter gene enhanced green-fluorescent protein (EGFP) in E1A-MEF. The resulting cell population was incubated with St42Rag2^−/−^ splenocytes followed by long-term culture at low density. Surviving clones expressing the EGFP reporter were expanded, and immune resistance was validated by probing clonogenic survival following incubation with St42Rag2^−/−^ splenocytes. Genomic DNA was isolated from validated clones, and integrated vector-encoded cDNA sequences were retrieved by polymerase chain reaction and sequencing (Supplementary Figure 2A). The vector DNA from one resistant clone encoded the cyclooxygenase 2/prostaglandin-endoperoxide synthase 2 (COX-2) sequence. Based on its reported role in colorectal cancer pathogenesis^20^ we selected COX-2 for further study. The full length human *COX-2* and murine *Cox-2* cDNAs were cloned into retroviral vectors and stably expressed in E1A-MEF to obtain E1A-Cox2-MEF (Supplementary Figure 2B). Following incubation with St42Rag2^−/−^ splenocytes E1A-Cox2-MEF showed markedly enhanced clonogenic survival as compared with control E1A-MEF (Figure 1a).

To validate COX-2 as an immune resistance factor *in vivo* we developed two models. In a model of adoptive cellular therapy we established E1A-MEF and E1A-Cox2-MEF fibrosarcomas by subcutaneous injection of 1 × 10^6^ cancer cells in the flank region of immunocompromised NOD/SCID mice. Palpable tumors developed within 3 days. Tumor-bearing mice received single intravenous injections of St42Rag2^−/−^ splenocytes (38 × 10^6^ cells in 150 *μ*l saline). A single intramuscular injection of recombinant murine interleukin-2 (rmuIL-2, 3 *μ*g) dissolved in 100 *μ*l saline and incomplete Freud's adjuvant was administered as adjuvans. Tumor growth and survival were monitored every 2 days. Mice bearing fibrosarcomas >15 mm in diameter were euthanized following German Animal Protection Law and institutional guidelines. Median survival of untreated NOD/SCID mice bearing E1A-MEF or E1A-Cox2-MEF fibrosarcomas was 22±2 days (median±S.E.M.) and 20±3 days, respectively (Figure 1b). Adoptive transfer of St42Rag2^−/−^ splenocytes prolonged median survival of E1A-MEF to 29±2 days (Hazard ratio (HR) 0.23, *P*=0.0027, log-rank test), thus supporting an antigen-specific tumor-suppressive activity of adoptively transfered TCR transgenic St42Rag2^−/−^ splenocytes *in vivo*. The median survival of identically treated NOD/SCID mice bearing E1A-Cox2-MEF fibrosarcomas was significantly shorter (median 27±4 days, HR 0.34, *P*=0.0347, log-rank test). It was comparable with fibrosarcoma-bearing mice not adoptively treated with splenocytes (Figure 1b).

In an immune surveillance model we subcutaneously injected 1 × 10^6^ E1A-MEF or E1A-Cox2-MEF cells into the flank region of St42Rag2^−/−^ mice. We expected that endogenous TCR transgenic T cells of these mice would impact on the outgrowth of E1A-positive fibrosarcomas. In fact, tumors from E1A-Cox2-MEF grew at a faster rate than those from E1A-MEF (Figure 1c). In summary, both models support a role for Cox-2 as mediator of resistance against tumor suppression by antigen-specific T cells *in vivo*.

### COX-2 mediated resistance depends on its catalytic activity and is immune-specific

**Figure 2:**
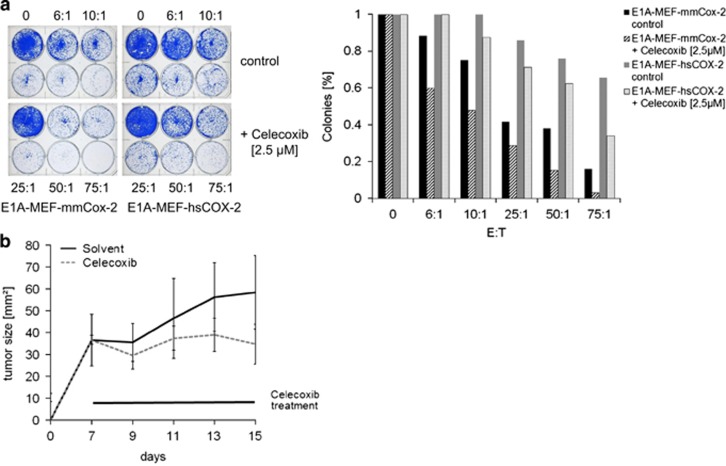
Cox-2-mediated resistance is dependent on its enzymatic activity and prostaglandin synthesis. (**a**) Celecoxib reduces the clonogenic survival of E1A-MEF expressing mmCox-2 or hsCOX-2 following incubation with St42Rag2^−/−^ splenocytes at indicated effector-to-target ratios. A representative photograph (left panel), and enumeration of colonies normalized to medium control are shown (right panel). (**b**) Tumor growth of MC-GP cells injected subcutaneously in the flanks of C57BL/6 mice. Animals were treated once daily with Celecoxib (10 mg/kg) or solvent by gavage. Mean values (±S.E.M.) of six mice per group are shown

COX-2 catalyzes the formation of prostaglandin H2 from arachidonic acid, which then matures into prostaglandins including prostaglandin E2 (PGE2). Prostaglandins mediate their biologic activities by engaging specific G-protein-coupled receptors.^21^ In keeping, significantly increased PGE2 levels were detected in cell culture supernatants from E1A-Cox2-MEF as compared with controls (Supplementary Figure 3A). The COX-2 selective inhibitor celecoxib effectively suppressed PGE2 release by cultured MEF (Supplementary Figure 3B). E1A-MEF expressing the murine or human COX-2 cDNA incubated with St42Rag2^−/−^ splenocytes showed reduced long-term clonogenic survival when celecoxib was added (Figure 2a). To assess the impact of COX-2 on a *de novo* antitumoral immune response we studied the formation and growth of murine MC-GP tumors in immune competent C57BL/6 mice. MC-GP is a murine fibrosarcoma cell line with high endogenous Cox-2 expression (Supplementary Figure 3C). Measurable tumors developed within 14 days after subcutaneous flank injection of 5 × 10^6^ MC-GP cells. Treatment with celecoxib (10 mg/kg body weight per day by gavage) effectively suppressed tumor formation in immune competent mice (Figure 2b), thus supporting a role of COX-2 activity in the suppression of antitumor immunity.

**Figure 3:**
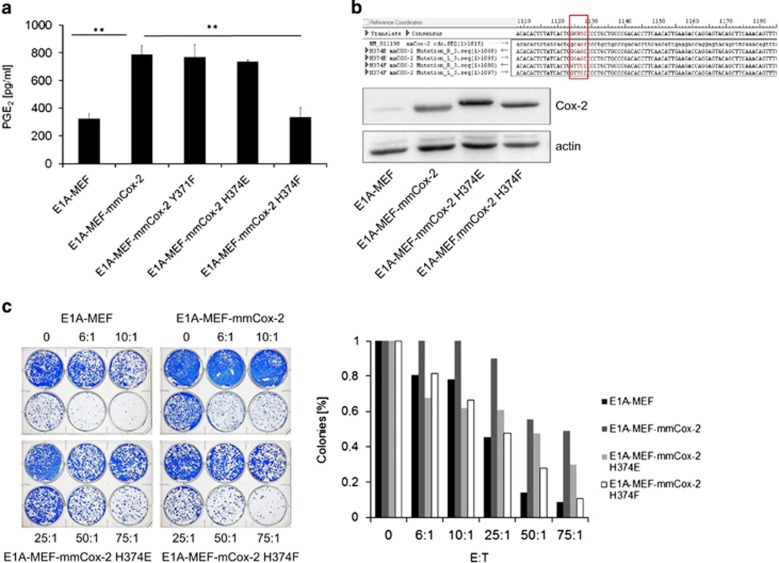
Enzymatically inactive COX-2 proteins are incapable of conferring immunoresistance against antigen-specific T cells. (**a**) Release of PGE2 into the cell culture supernatant by E1A-MEF, E1A-Cox2-MEF and E1A-MEF expressing various mmCox-2 mutants. Mean values (+S.D.) of triplicate experiments are given (**P<0.01, *t*-test). (**b**) Site-directed mutagenesis of codon 374 of the murine Cox-2 cDNA (upper panel). The expression of wild type Cox-2 and Cox-2 mutants was confirmed by immunoblotting. Actin served as loading control (lower panel). (**c**) Clonogenic survival of E1A-MEF, E1A-Cox2-MEF and E1A-MEF expressing the mmCox-2^H374E^ or mmCox-2^H374F^ mutants following incubation with St42Rag2^−/−^ splenocytes at indicated effector-to-target ratios. A representative photograph (left panel), and enumeration of colonies normalized to medium control are shown (right panel)

As celecoxib may have off-target effects or could directly impact on T-cell function and tumor cell proliferation we decided to apply a genetic model for further study of COX-2 function in treatment resistance of cancer. Surprisingly, E1A-MEF expressing the murine Cox-2^Y371F^ mutant^22^ released comparable amounts of PGE2 as E1A-Cox2-MEF (Figure 3a). Accordingly, we reasoned that the C-terminal catalytic site of Cox-2 might be crucial for prostaglandin synthesis in MEF. We therefore introduced base exchanges at codon 374 of the murine *Cox-2* cDNA by site-directed mutagenesis and stably expressed the resulting Cox-2^H374E^ and Cox-2^H374F^ mutants in E1A-MEF (Figure 3b). In particular E1A-Cox2^H374F^-MEF released less PGE2 into the cell culture supernatant (Figure 2a), which coincided with impaired immune resistance when cocultured with St42Rag2^−/−^ splenocytes (Figure 3c).

COX-2 is reported to prevent apoptosis by engaging mitogen-activated protein kinases (MAPK), protein kinase A (PKA) and phosphoinositide-3-kinase (PI3K) signaling.^23^ This leads to increased expression of anti-apoptotic BCL-2 family proteins and transactivation of the proliferator-activated receptor (PPAR)-delta.^24, 25^ Deregulated COX-2 expression is also thought to reduce the pool of intracellular arachidonic acid and consequently ceramide, thus raising the threshold at which cancer cells undergo apoptosis.^26^ Against this background we compared the susceptibility of E1A-MEF, E1A-MEF expressing the EGFP reporter and E1A-Cox2-MEF to various apoptotic stimuli. Over a wide range of concentrations and doses COX-2 conferred no resistance to apoptosis induced by staurosporine (Supplementary Figure 4A), paclitaxel (Supplementary Figure 4B), etoposide (Supplementary Figure 4C) or UV radiation (Supplementary Figure 4D). These results argued against a general anti-apoptotic activity of COX-2 at least in our experimental systems. This also argued against COX-2 acting at the level of BCL-2 family members, which provide cross-resistance to apoptosis mediated by perforin-dependent immune effector mechanisms.^27, 28^

### COX-2 expression induces immunosuppressive factors and blunts interferon-gamma release by T cells exposed to antigen-presenting cells

We reasoned whether local COX-2 activity impacts on the composition of tumor infiltrating immune cell subsets. MC-GP tumors with high endogenous COX-2 expression were established in immune competent C57BL/6 mice that were treated or not treated with celecoxib. Immunohistochemical analyses of explanted tumors revealed an immune infiltrate consisting of CD90.2-positive T cells, CD11b/Gr1-positive myeloid-derived suppressor cells, and CD4/Foxp3-positive regulatory T cells, which was not altered by COX-2 inhibition (Supplementary Figure 5). Thus, in this model COX-2 seems to impact on immune cell function rather than immune cell recruitment.

**Figure 4:**
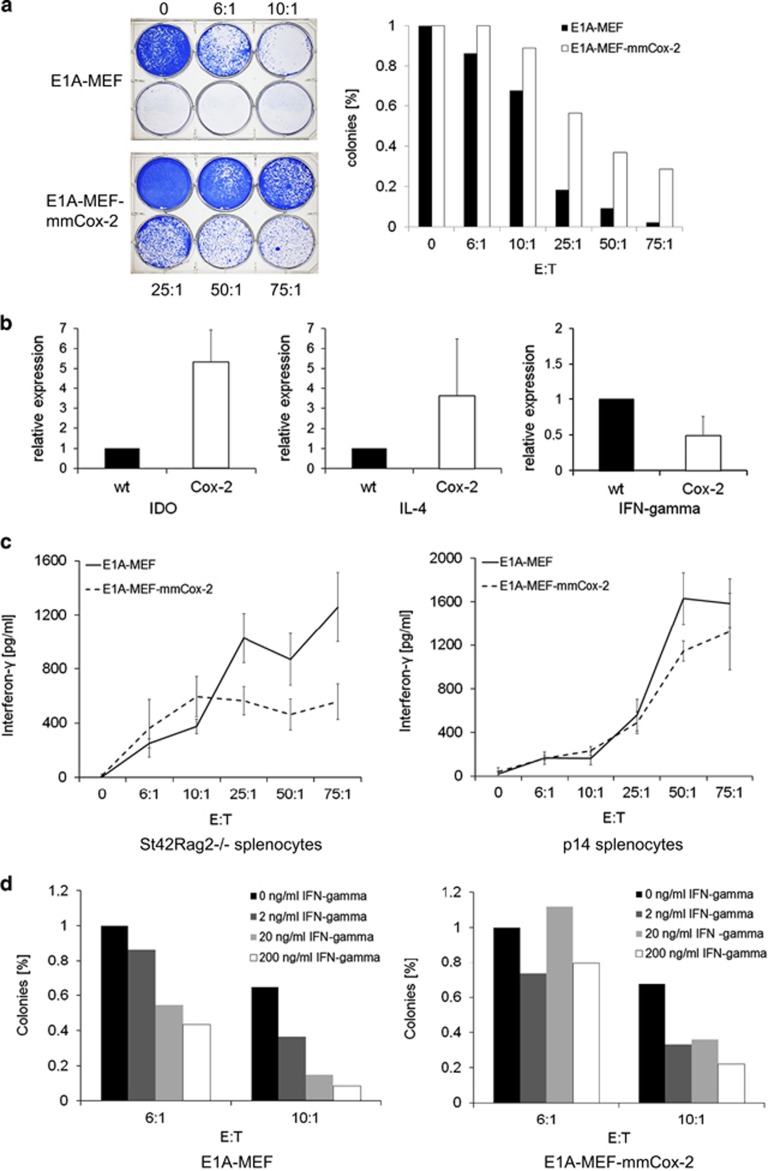
COX-2 blunts the release of interferon-gamma by antigen-activated T cells. (**a**) Clonogenic survival of E1A-MEF and E1A-Cox2-MEF loaded with the LCMV gp33 peptide following incubation with gp33-specific p14 splenocytes. A representative photograph (left panel), and enumeration of colonies normalized to medium control (mean values) of three independent experiments each are shown (right panel). (**b**) Transcriptional expression levels of *Il-4* and *Ido1* are increased in tumors overexpressing Cox-2 while the expression of interferon-gamma is down regulated. *Ex vivo* RT-PCR analysis of tumors from E1A-MEF and E1a-Cox-2-MEF using specific primers for *Il-4*, *Ido1* and interferon-gamma. Expression levels were normalized to actin. (**c**) Release of interferon-gamma by St42Rag2^−/−^ splenocytes (left panel) or p14 splenocytes (right panel) incubated with E1A-MEF (solid lines) or E1A-Cox2-MEF (dashed lines). Prior to incubation with p14 splenocytes the MEF were loaded with LCMV gp33 peptide. Mean values (± SEM) of three independent experiments. (**d**) Clonogenic survival of E1A-MEF (left panel) or E1A-mmCox2-MEF (right panel) following incubation with St42Rag2^−/−^ splenocytes at indicated effector-to-target ratios in the presence of increasing concentrations of recombinant murine interferon-gamma (IFN-gamma). Colonies were enumerated and normalized to the colony number in the absence of IFN-gamma

To explore whether COX-2-mediated immune resistance is of general importance we turned to another defined T-cell receptor system. Transgenic C57BL/6 p14 mice harbor a TCR directed against the lymphocytic choriomeningitis virus (LCMV) antigen gp_33–41_ (gp33) presented in the context of H2-Kb.^29^ When E1A-MEF and E1A-Cox2-MEF were loaded with the gp33 peptide and incubated with p14 splenocytes, COX-2 expression again mediated significantly increased clonogenic survival of cancer cells (Figure 4a).

Next we sought to further define the mechanism of COX-2-mediated immune resistance. E1A-MEF and E1A-Cox2-MEF fibrosarcomas established in immune competent C57BL/6 mice were explanted and homogenized to study intratumoral cytokine expression. Interestingly, COX-2-fibrosarcomas exhibited increased expression of *Il4* and *Ido1* encoding interleukin-4 and Ido, whereas the expression of *Ifng* encoding interferon-gamma was significantly reduced (Figure 4b). This argues that high local COX-2 expression causes an immunosuppressive milieu and impairs T cells' effector functions mediated by interferon-gamma.

To further support this observation we measured the release of interferons by TCR transgenic splenocytes incubated with their respective cellular targets in relation of COX-2 expression. While the levels of interferon-alpha remained unchanged (not shown), St42Rag2^−/−^ splenocytes incubated with E1A-Cox2-MEF as well as p14 splenocytes incubated with gp33 peptide-loaded E1A-Cox2-MEF exhibited a blunted release of interferon-gamma as compared with splenocytes incubated with their respective control targets (Figure 4c). Moreover, the additition of exogenous interferon-gamma reduced clonogenic survival of E1A-Cox2-MEF incubated with St42Rag2^−/−^ splenocytes at a higher effector-to-target ratio (Figure 4d). In summary, immunological control of cancer cells with high COX-2 expression by antigen-specific T cells may be less effective because of increased local release of IL4 and IDO and blunted interferon-gamma secretion by antigen-activated cytotoxic T cells.

## Discussion

Clinically applied immunotherapeutic strategies for malignant diseases are manifold. The adoptive transfer of HLA-matched allogeneic cellular immune effectors during allogeneic hematopoietic stem cell transplantation or donor lymphocyte infusion is thought to suppress the host hematopoiesis (including the leukemic clones) by a sustained alloresponse directed against disparate minor histocompatibility antigens.^30, 31^ These allogeneic cellular therapies have evolved as standard of care for medically fit patients with high-risk leukemias and available HLA-matched stem cell donor. In contrast, antigen-specific immunotherapies in the autologous settings are clinically less established. Recently, an autologous cellular vaccine was shown to confer a modest survival benefit in patients with castration-resistant prostate cancer.^32^ HLA-A*0201-positive patients with metastastic renal cell cancer treated with the multipeptide vaccine IMA901 appeared to derive a higher benefit if an effective immune response was raised.^33^ Such studies sustain the hopes that the principle of activating an antigen-specific autologous immune response will eventually provide clinically meaningful improvements for a larger group of cancer patients. More recently, the so-called 'immune checkpoint inhibitors' have evolved as another, antigen-independent immunotherapeutic principle with high potential. Antibodies blocking CTLA-4 or interfering with the PD-1/PD-L1 interaction have proven clinical efficacious in patients with metastatic malignant melanoma.^34, 35, 36^ Moreover, early studies have suggested clinical activity in cancers of the lung, kidney and other entities.^37, 38^ Although these examples follow different strategies to evoke a cancer-directed cellular immune response, they all rely on the susceptibility of cancer cells to the cellular immune effector mechanisms.

Against this background it is important to identify and dissect cell-autonomous mechanisms that impact on the response of a cancer cell to the encounter with activated cytotoxic T cells. Such studies may help to nominate predictive biomarkers that can be applied to enrich patient populations with higher likelihood of response or resistance to a given immunotherapeutic intervention. Moreover, such resistance pathways may serve as targets for pharmacotherapies aiming to overcome cancer cell-autonomous immune resistance or to enhance the efficacy of cancer immunotherapies. Previously, we and others have shown that the expression of anti-apoptotic regulators confer resistance to the tumor-suppressive activity of activated cytotoxic T cells and perforin-dependent effector mechanisms.^28, 39, 40, 41, 42^ Also, oncogenic growth and survival signals were found to modulate the susceptibility of cancer to potent alloreactive T cells^19, 27^ or direct and indirect antibody-mediated tumor suppression *in vitro* and *in vivo.*^43, 44^ These studies identified mechanisms that equally protect against immunotherapies and conventional cytotoxic therapies such as chemotherapy or radiation. Selection for resistance to one of these modalities thus may lead to 'cross-resistance' against others. Here we describe an example of a cell-autonomous resistance mechanism that is specific for cellular immune effectors. COX-2 expression decreases the sensitivity of cancer cells to activated antigen-specific T cells by inducing the expression of IL4 and IDO, and by modulating the release of interferon-gamma by activated T cells. The induction and release of interferons by T cells is triggered by antigen-specific TCR activation.^45^ It is crucially involved in the control if virally infected host cells.^46, 47^ Elaborate studies support a role for interferon-gamma in T-cell-mediated tumor elimination *via* direct and indirect mechanisms.^48, 49, 50^ In our experimental systems COX-2 failed to mediate cross-resistance to generic cytotoxic insults such as DNA damage or kinase inhibition. Nevertheless, it is entirely plausible that cancer cells are selected for COX-2 expression for biological activities in addition to immune resistance. A plethora of mechanistic studies in particular in colorectal cancer models describe how COX-2 may impact on cancer cell-autonomous proliferative and survival signals as well as the microenvironment.^51^ In this context our present findings add important aspects in support of a selective advantage of cancer cells expressing COX-2 in tumor development and progression. By suppressing interferon-gamma expression and release from antigen-specific T cells, and locally enhanced IL-4 and IDO levels COX-2 may contribute to a tumor-permissive milieu. This adds to reported immunosuppressive mechanisms of prostaglandins such as the induction of myeloid-derived suppressor cells through COX2-2/PGE2.^16, 52^ This provides a rationale for therapeutic targeting of COX-2 expression and stress-induced prostaglandin synthesis to boost immune surveillance and immunotherapy of cancer. This may, however, become a more complex issue than just treating with currently available COX-2 inhibitors as these might have off-target effects on immune cells and the microenvironment that could counterbalance their activity as enhancers of susceptibility to immune elimination.

## Materials and Methods

### Plasmids, antibodies and reagents

The complete cDNAs of murine *Cox-2* and human *COX-2* were cloned into the retroviral expression vector pLPCX. All inserts were verified by sequencing. Immunoblotting was performed using primary antibodies against actin (C4, MP Biomedicals, Santa Ana, CA, USA) and COX-2 (abcam, Cambridge, UK). Prostaglandin E_2_, celecoxib, staurosporine and incomplete Freund's Adjuvant were purchased from Sigma (St. Louis, MO, USA). Recombinant murine interferon-gamma and interleukin-2 were obtained from Chemicon (Millipore, Billerica, MA, USA). Etoposide and paclitaxel were obtained from the Pharmacy of the University Hospital Essen. The LCMV gp33 peptide was purchased from Neosystems (Straßbourg, France).

### Cells and cell culture conditions

Murine embryonic fibroblasts were generated from C57BL/6 mice and were transformed with two cooperating oncogenes, H-Ras and E1A or MYC, as described previously.^28^ Murine MC-GP cells (purchased from ATCC) and MEFs were maintained in Dulbecco modified Eagle medium (DMEM) supplemented with fetal bovine serum, l-glutamine, penicillin and streptomycin (Gibco, Carlsbad, CA, USA).

Cytotoxic T cells directed against the adenovirus E1A oncoprotein (amino acids 234-243 presented by H2-D^b^) were obtained from TCR transgenic St42*Rag2*^−/−^ C57BL/6 mice.^53, 54^ Cytotoxic T cells directed against the lymphocytic choriomeningitis virus (LCMV)-specific peptide gp33 (amino acids 33-41) were obtained from TCR transgenic p14 mice (C57BL/6 J.B6D2F2-TgN(^H2Kb^-TCRV*β*8.1).^55^ Spleens were isolated and strained through a micrometer mesh to obtain single-cell suspensions. Red blood cells were lysed with ammonium chloride solution. The resulting cell suspension was filtered through a cell strainer (40 *μ*m), centrifuged at 1400 r.p.m. for 5 min, and resuspended in fresh media.

To study tumor suppression by E1A-specific T cells *in vitro*, 5000 adherent MEFs were incubated in 96-well plates with St42*Rag2*^−/−^ splenocytes at various effector-to-target (E:T) ratios in the presence of IL-2 (120 U/ml). To study cytotoxicity mediated by p14 splenocytes E1A-MEF were preloaded with gp33 peptide (5 *μ*g/ml) for 90 min. After 24 h splenocytes were removed, the MEF targets were washed twice with phosphate-buffered saline, harvested and seeded at single-cell density in six-well plates. Following an average culture period of 5 days the resulting colonies were fixed with ethanol and stained with brilliant-blue for documentation and enumeration.

The cDNA library screen was conducted as previously described^19^ using E1A-MEF as targets and St42*Rag2*^−/−^ splenocytes as effectors.

### Prostaglandin and interferon measurements

The concentration prostaglandin E2 in cell culture supernatants was measured by the Prostaglandin E2 EIA Kit—Monoclonal (Cayman Chemicals, Ann Arbor, MI, USA) following the manufacturer's instructions. The release of interferon-gamma and interferon-alpha by T cells cocultured with MEF was quantified using the Mouse IFN-gamma Platinum ELISA (eBioscience, San Diego, CA, USA) and VeriKine Mouse Interferon Alpha ELISA Kit (PBL Interferon Source, Piscataway, NJ, USA).

### *In vivo* tumor models

Immune compromised NOD/SCID mice received single subcutaneous injections of 1 × 10^6^ MEF in the left flank. Tumor development was monitored by palpation and usually could be detected after 3 days. Tumor-bearing mice were adoptively treated with 38 × 10^6^ unprimed St42*Rag2*^−/−^ splenocytes resuspended in saline administered as single tail vein injection. Recombinant murine IL-2 (3 *μ*g) was dissolved in 50 *μ*l incomplete Freund's adjuvant and 50 *μ*l saline and administered as adjuvans by a single intramuscular injection.

In the immune surveillance model TCR transgenic St42*Rag2*^−/−^ mice received single subcutaneous injections of 1 × 10^6^ E1A-MEF and E1A-Cox2-MEF, respectively. To study an endogenous *de novo* antitumoral immune response C57BL/6 mice received single subcutaneous flank injections of 5 × 10^6^ MC-GP cells, E1A-MEF or E1A-Cox2-MEF.

Tumor development was detected by palpation and growth was measured bidimensionally using a caliper. Mice were euthanized once the tumor size had exceeded 15 mm.

To study intratumoral cytokine expression, fibrosarcomas were explanted and homogenized (MagNA Lyser, Roche, Grenzach-Wyhlen, Germany) followed by RNA extraction (RNeasy Mini Kit, QIAgen, Hilden, Grenzach-Wyhlen). Expression of *Il4*, *Ido1* and *Ifng* was quantified by reverse transcription (Transcriptor High Fidelity Kit, Roche) and real-time polymerase chain reaction on a LC480 II system (Roche), using specific primers (QIAgen). Tumor-infiltrating immune cells were characterized in snap-frozen tumor tissue (Tissue Tek, Sakura, Japan). Cryosections were fixed with acetone followed by blocking with phosphate-buffered saline and 2% fetal bovine serum. Following immunohistochemical staining images were acquired on a confocal microscope (Leica TCS SP8, Leica, Wetzlar, Germany). Antibodies were CD90.2-APC, CD4-APC, Foxp3-PE, CD11b-PE and Gr1-FITC (ebiosciences). Nuclei were counterstained with DAPI (Sigma, Seelze, Germany).

All animal studies were conducted in compliance with German Animal Protection Law and institutional guidelines, and were approved by the responsible regulatory authority (LANUV NRW).

## Abbreviations

BCL-2: B-cell lymphoma 2
COX-2: cyclooxygenase 2
CTLA-4: cytotoxic T-lymphocyte antigen 4
E1A: adenovirus early region 1 A
EGFP: enhanced gene green-fluorescent protein
gp33: glycoprotein 33
IDO: Indoleamine 2,3-dioxygenase
LCMV: lymphocytic choriomeningitis virus
MAPK: mitogen-activated protein kinases
MEF: murine embryonic fibroblast
MHC: major histocompatibility complex
PD-1: programmed death 1
PD-L1: programmed death-ligand 1
PGE2: prostaglandin E2
PI3K: phosphoinositide-3-kinase
PKA: protein kinase A
PPAR-delta: proliferator-activated receptor delta
Rag2: recombination activating gene 2
TCR: T-cell receptor
