# Supplementary figures and images for: Functional expression cloning identifies COX-2 as a suppressor of antigen-specific cancer immunity
Source: Cell Death Dis. 2014 Dec 11;5(12):e1568–. doi: 10.1038/cddis.2014.531 (PMC4649842; doi:10.1038/cddis.2014.531)

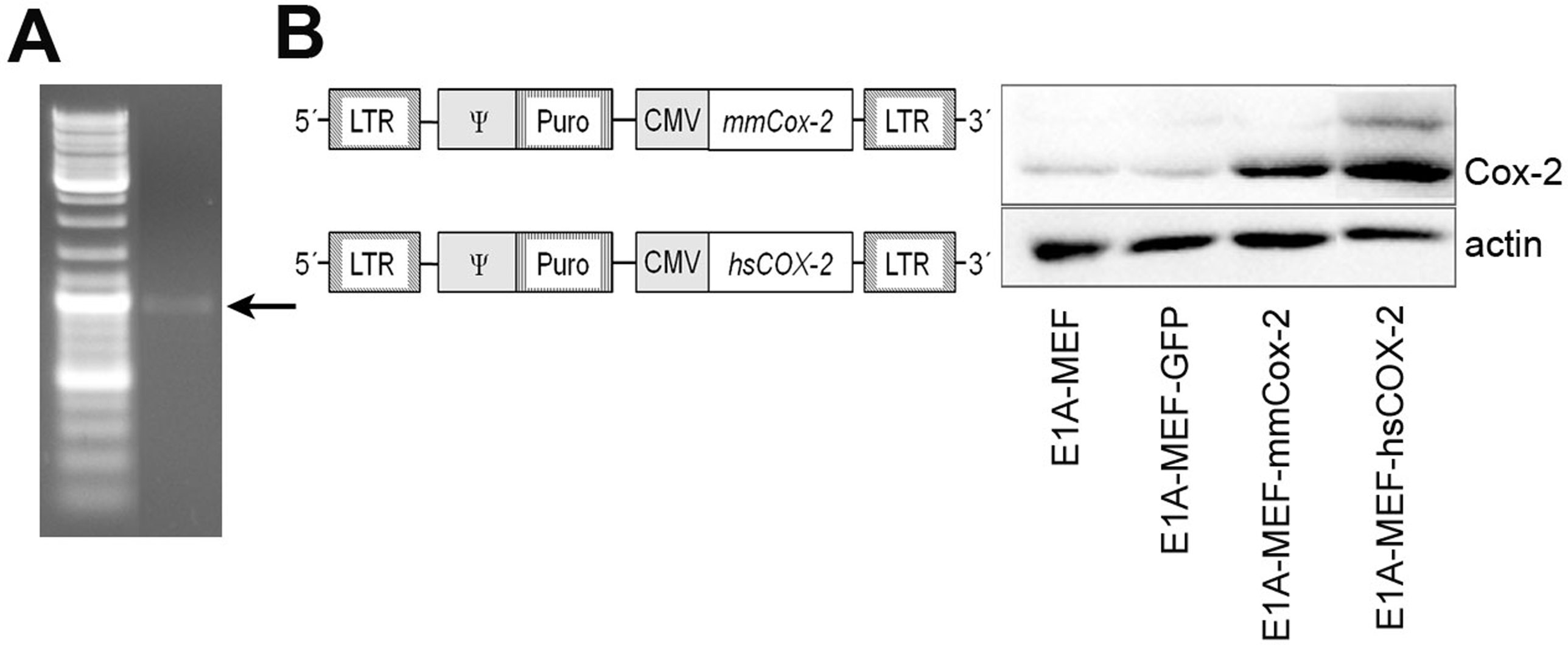

Supplement: Supplementary Figure 2 [file cddis2014531x2.tif]

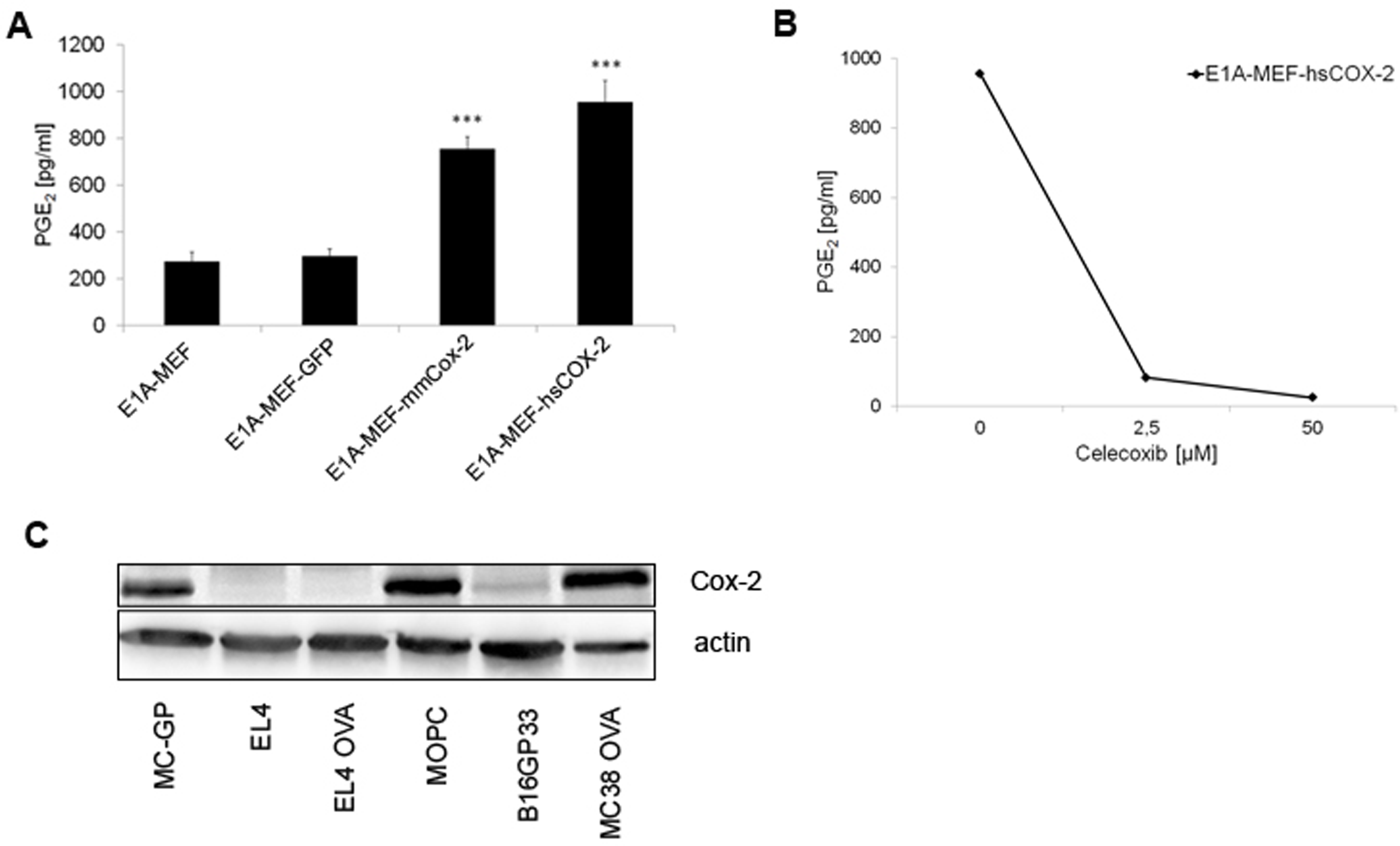

Supplement: Supplementary Figure 3 [file cddis2014531x3.tif]

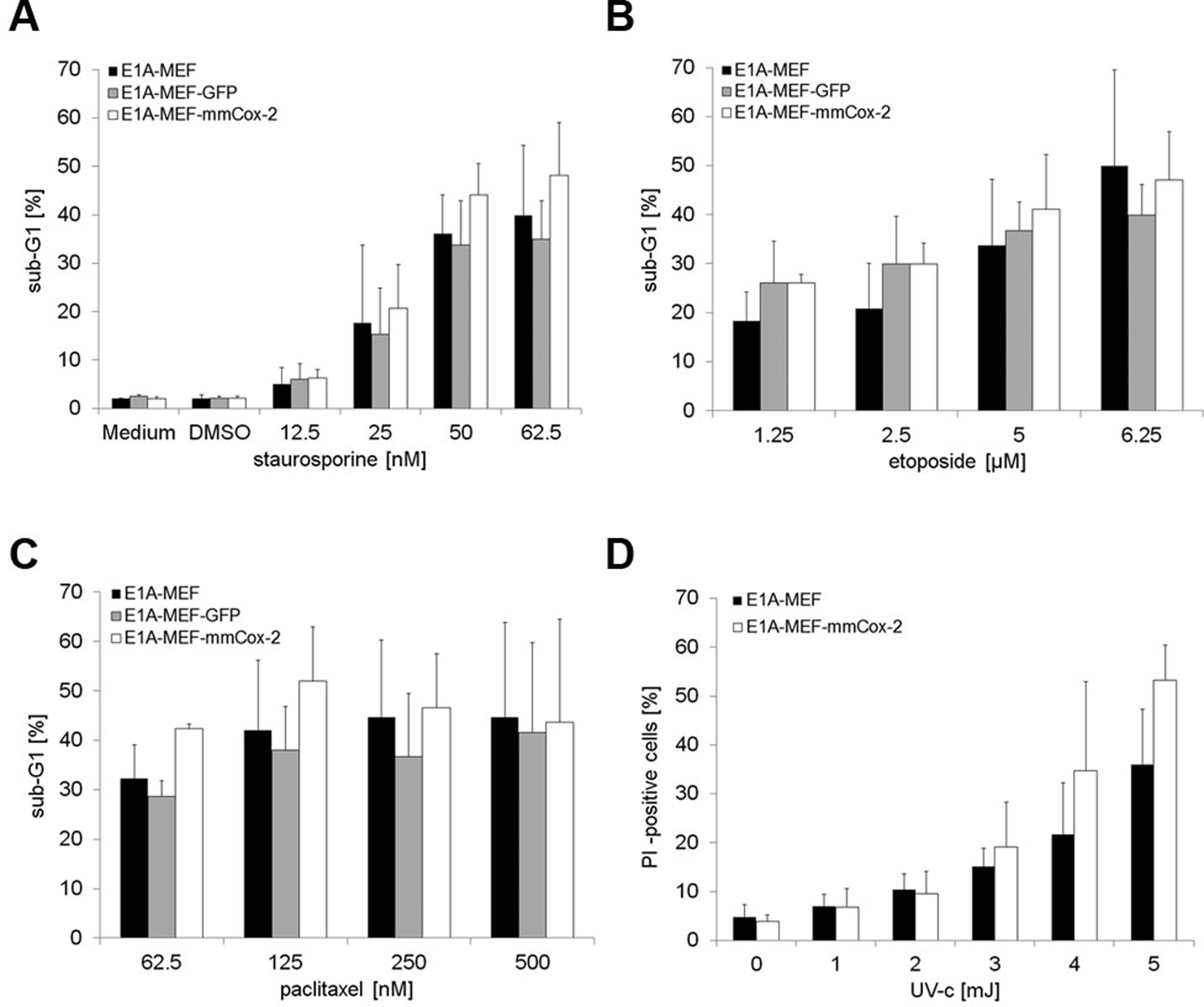

Supplement: Supplementary Figure 4 [file cddis2014531x4.tif]

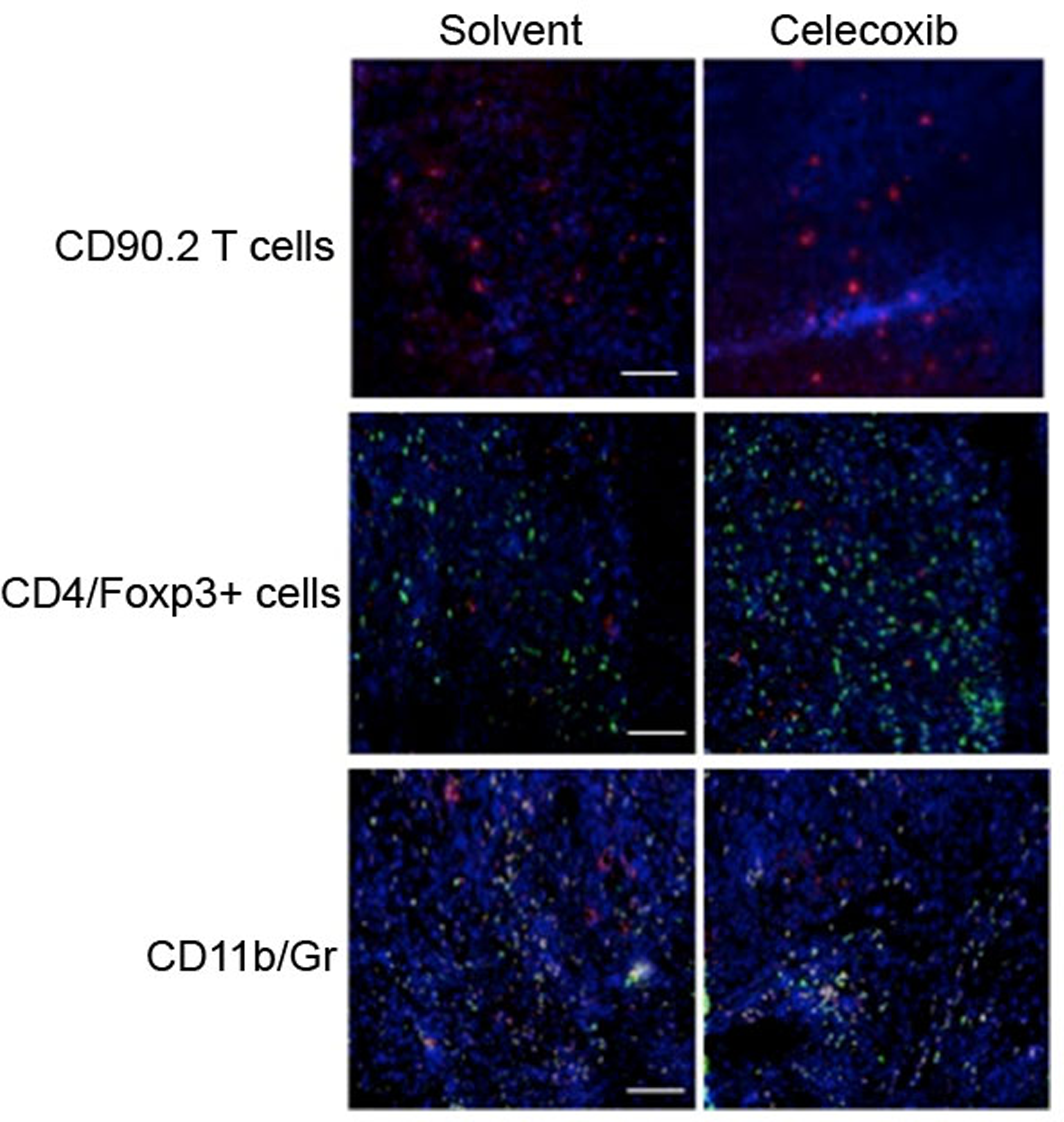

Supplement: Supplementary Figure 5 [file cddis2014531x5.tif]
