# Supplementary material for: Functional expression cloning identifies COX-2 as a suppressor of antigen-specific cancer immunity
Source: Cell Death Dis. 2014 Dec 11;5(12):e1568–. doi: 10.1038/cddis.2014.531 (PMC4649842; doi:10.1038/cddis.2014.531)
Supplement: Supplementary Figure Legends [file cddis2014531x6.doc]

**Supplementary Figure Legends**

**Supplemental Figure 1**

**Antigen-specific tumor suppression of E1A-MEF but not Myc-MEF incubated with St42Rag2^-/-^ splenocytes**

Representative photograph of long-term colony formation following incubation of E1A-MEF (left) or Myc-MEF (right) with St42Rag2^-/-^ splenocytes at indicated effector-to-target ratios.

**Supplemental Figure 2**

**Identification of COX-2 as resistance factor against tumor suppression by antigen-specific T cells**

(A) PCR product of vector-encoded cDNA in a resistant clone. Sequencing revealed the human COX-2 cDNA.

(B) Retroviral vector constructs (left panel) for expression of the human *COX-2* cDNA and the murine *Cox-2* cDNA. Transgene expression was confirmed by immunoblotting using primary antibodies against COX-2, and actin as loading control (right panel).

**Supplemental Figure 3**

**Prostaglandin E2 synthesis and release by E1A-MEF ectopically overexpressing murine or human COX-2 and endogenous overexpression of COX-2 by murine MC-GP cells**

(A) Release of prostaglandin E2 (PGE2) into the cell culture supernatant by E1A-MEF, E1A-MEF expressing the EGFP reporter gene (E1A-MEF-EFGP), and E1A-MEF expressing the murine (mm) Cox-2 or human (hs) COX-2 cDNA. Mean values (+ SD) of triplicate experiments are given (***p < 0.001, t-test)

(B) The COX-2 inhibitor celecoxib (2.5 and 50 µM) effectively blocks the release of PGE2 by E1A-Cox2

MEF (mean values) of triplicate experiments are given.

(C) Cox-2 protein expression was detected by immunoblot in MC-GP cells as well as various other cell lines (i.e. EL4 and EL4OVA, MOPC, B16GP33 and MC38OVA). Antibodies against COX-2, and actin as loading control were applied.

**Supplemental Figure 4**

**COX-2 fails to protect E1A-MEF against various apoptotic stimuli**

Parental E1A-MEF, E1A-MEF expressing the EGFP reporter gene (E1A-EGFP-MEF), and E1A-Cox2-MEF were treated with staurosporine (A), etoposide (B), paclitaxel (C), or UV-C radiation (D) at the indicated concentrations and doses. Apoptotic cell death was quantified by flow cytometry to detect subgenomic DNA after 24 hours (staurosporine), 48 hours (etoposide) and 72 hours (paclitaxel). Mean values (+ SD) of three independent experiments are given.

**Suppl. Figure 5**

**Immunohistologic analyses of immune cell infiltration in MC-GP tumors**

Shown are immunohistologic analyses of tumor samples from celecoxib- or solvent-treated mice three weeks after s.c. tumor inoculation. Pictures show representative results (6 mice per group). Anti-CD90.2, anti-CD11b and anti-Foxp3 signals are shown in red, anti-CD4 and anti-Gr1 signals are shown in green and DAPI stains in blue. Scale bar = 100µm.
